# Ureteral tumor in an ectopic duplex system: a case report

**DOI:** 10.1186/s13256-019-1974-2

**Published:** 2019-03-08

**Authors:** Omar Karray, Hassen Khouni, Mahdi Charfi, Rami Boulma, Mehdi Debaibi, Rym Makhlouf, Karim Bargaoui, Oumeima Nessej, Azza Seridi, Slim Fourti, Mohamed Habib Bouhaouala, Adnene Chouchene

**Affiliations:** 1 Urology Unit, Interior Security Forces Hospital, La Marsa, Tunisia; 2 Medical Imagery Department, Interior Security Forces Hospital, La Marsa, Tunisia; 3 General Surgery Department, Interior Security Forces Hospital, La Marsa, Tunisia

**Keywords:** Ureter abnormalities, Ureteral neoplasms, Nephroureterectomy

## Abstract

**Introduction:**

Ureteral ectopia is a rarely observed anomaly. It may be totally asymptomatic. An association with a duplex system is exceptional. Diagnostic and therapeutic approaches are challenging. Carcinologic surgery must consider the anatomic variant, mainly related to the ectopic site of the ureteral orifice.

**Observation:**

We report a case of a ureteral urothelial carcinoma in a North African 52-year-old male patient, in a right duplex system. Radiological explorations concluded a non-functional upper right kidney.

A suspect mass was observed in the lumbar part of the ureter of the right upper system. The meatus of the tumorous ureter ended in the right lobe of the prostate. A right hemi-nephro-ureterectomy was performed. A histological examination concluded a pT2G2 urothelial carcinoma.

**Conclusion:**

Even if malignancy is rarely observed in ureteral ectopia, it should be evoked mainly in cases of hematuria with risk factors for urothelial tumors.

## Introduction

Ureteral ectopia is an extremely rare congenital malformation. Certain forms remain asymptomatic for a long time and sometimes go unnoticed. Advances in the precision of radiological investigations enable a better description of anatomical variations. Malignant degeneration is an exceptional phenomenon. The stakes are double: diagnostic and therapeutic. A curative surgery should be preceded by a precise cartography of the urinary tract that describes, in particular, the distal anastomosis of ectopic ureter.

## Case presentation

A 52-year-old North African man, who smokes 30 packs of cigarettes per year, consulted for intermittent total and clotting hematuria for 2 weeks, without other functional complaints. He has no particular medical history. He was operated on at the age of 5 for bilateral cryptorchidism.

A physical examination was normal. There was no localized tenderness and no palpable mass in the lumbar fossae. His prostate was painless, without suspect lesions. His hemoglobin level was 13 g/dl. His platelet level, homeostasis, and renal function were normal. Urine analysis was sterile, with red blood cells in direct examination. Prostate-specific antigen was at 1.09 ng/ml.

On ultrasound, there were no suspect bladder lesions. An enlarged hypoechoic mass of the upper pole of his right kidney, extended by a dilated ureter, was observed. A suspect hypovascularized intraluminal papillary tumor in the right lumbar ureter was also noticed. The lower pole of his right kidney had a normal aspect, and was extended by a non-dilated ureter, suspicious for a double excretory system.

**Fig. 1:**
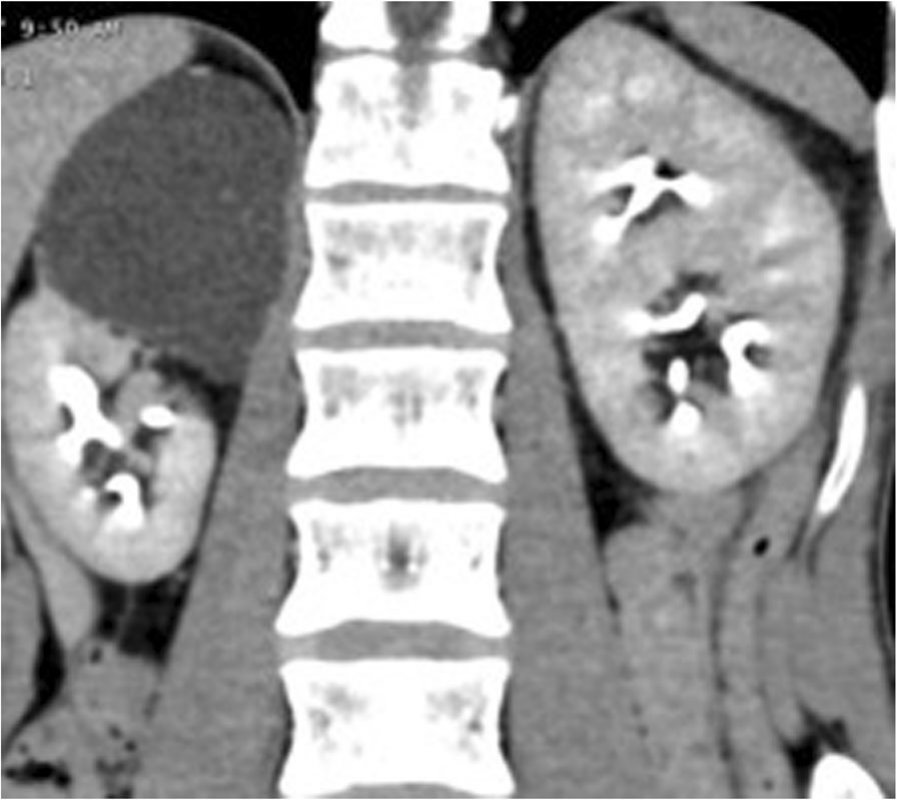
Uro-scan: right ureteral duplicity. The upper urinary system of the right kidney is destroyed

**Fig. 2:**
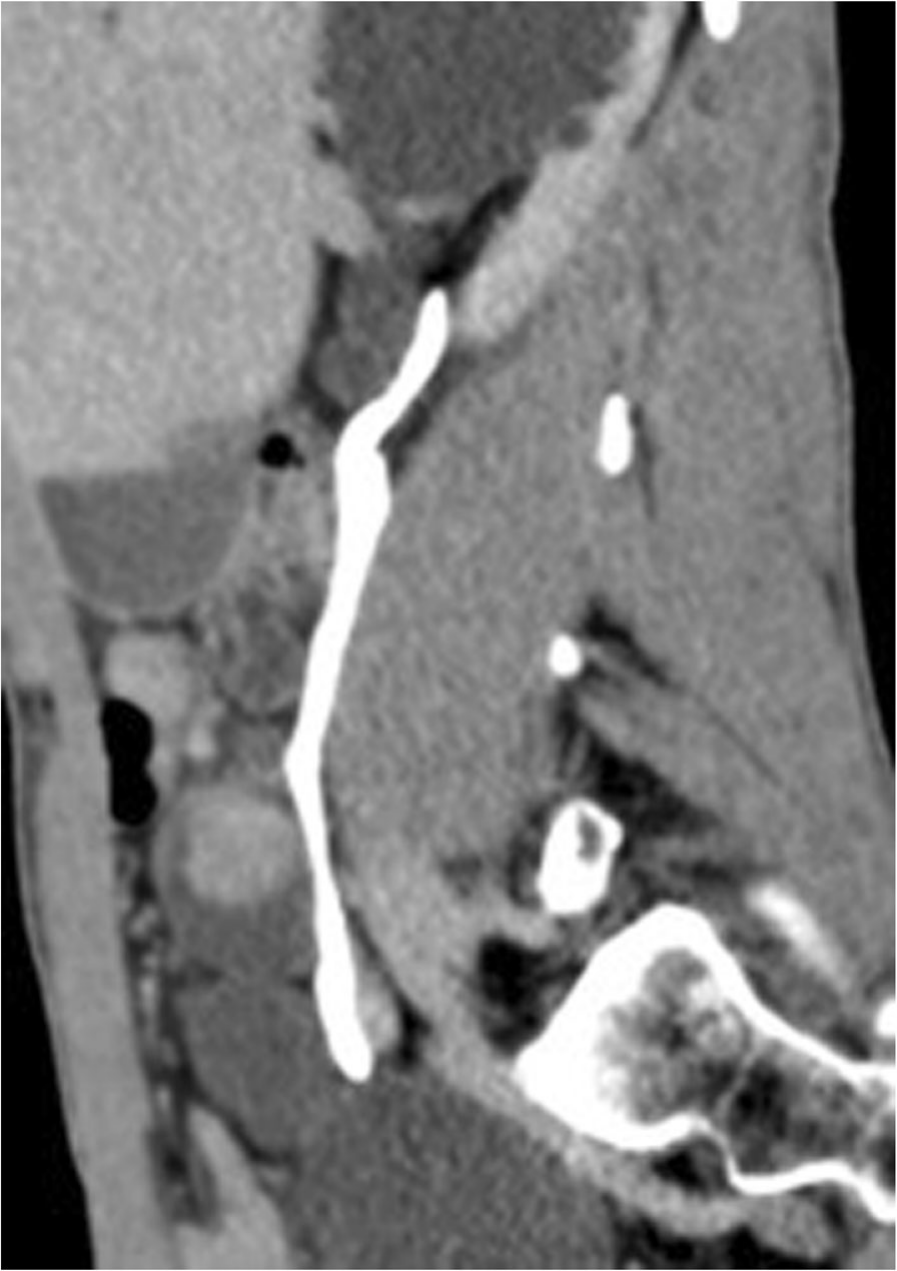
Uro-scan: a suspect 20 mm mass in a dilated ureter draining the upper system of the right urinary tract

**Fig. 3:**
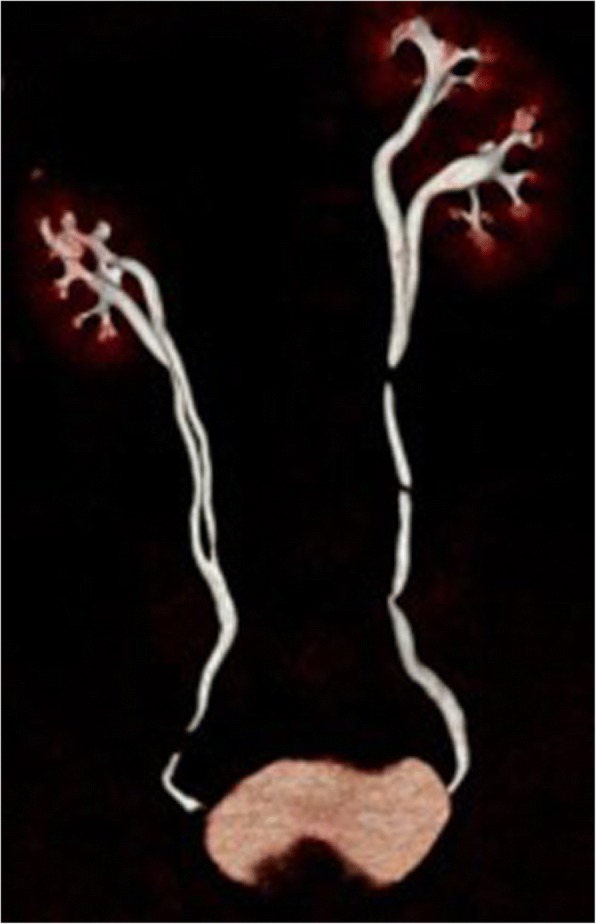
Uro-scan (reconstruction features): the ureter draining the lower pole of the right kidney is bifid

A computed tomography (CT) scan confirmed the right ureteral duplicity with a destroyed upper pole (Fig. 1). The ureter of the upper system was dilated, with an endoluminal tumor of 20 mm, at the height of the L4–L5 disc, partially calcified and highly enhancing following contrast injection (Fig. 2). The lower system had conserved secretory and excretory functions, and was itself a seat of ureteral bifidity with a common terminal ureteral segment coming into the bladder. There was also a left ureteral bifidity, with two systems secreting and excreting normally, without suspect lesions of the excretory tract (Fig. 3).

**Fig. 4:**
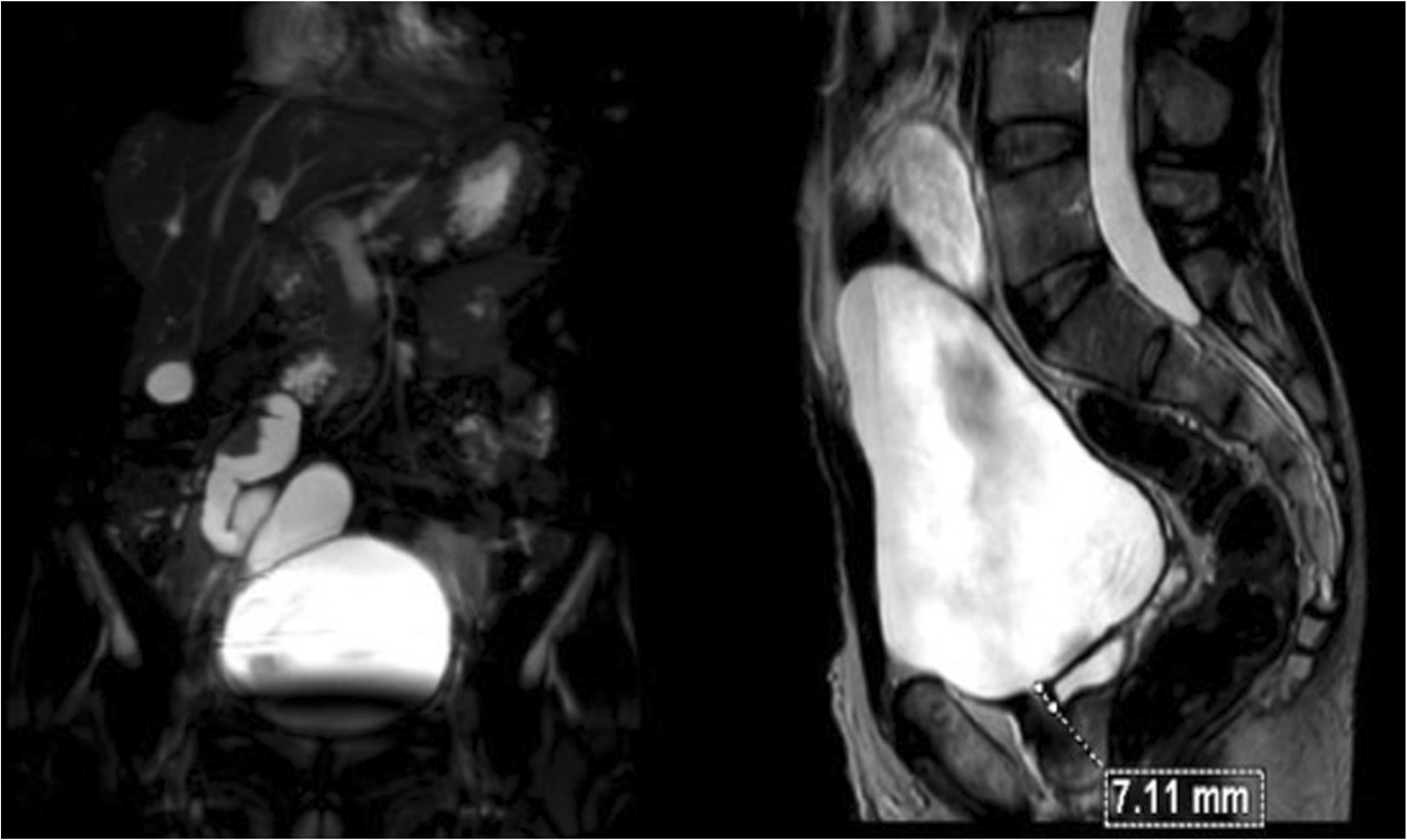
Uro-magnetic resonance imaging: tumorous ureter ends in the right prostatic lobe

An MRI was performed to define precisely the tumorous ureter orifice. The drainage ended in the prostatic right lobe without any parenchymal lesions observed (Fig. 4).

Cystoscopy under anesthesia was normal. The two meatuses were of normal seat.

A right ureteroscopy confirmed the ureteral bifidity, with no macroscopically suspect lesions. The ureter ending in the right upper tract was not visible at the prostatic urethra.

Our patient underwent surgery by a double incision: a right posterolateral lumbar incision, followed by a right iliopubic incision. A hemi-nephrectomy of the upper system of the right urinary tract was performed, going as low as possible on the ectopic ureter.

The iliopubic approach enabled us to dissect and release the ureter throughout its length, up to the prostatic apex. The section of the ureter was as close as possible to the prostatic capsule. An aspirative drainage of the lumbar fossa and the retropubic space was left at the end of the intervention.

His postoperative course was uneventful. The drains were withdrawn on the third postoperative day. He left the hospital on the fifth postoperative day.

A histological examination concluded a unifocal infiltrative transitional cell carcinoma, classified pT2G2. There was no lymphovascular invasion or vascular emboli. The lower ureteral section was free from any tumor proliferation.

At 2-year follow-up, there were no clinical or radiological signs of local or distant recurrence. He did not present hematuria recurrence, urinary troubles, or sexual troubles. A thoraco-abdomino-pelvic scan performed every 6 months during follow-up was without anomalies.

## Discussion

Ureteral ectopia is a rarely observed condition. It is more frequent within women and on the right side, with a sex ratio of 1:12 [1]. This malformation is related to a low insertion of the ureteral bud on the mesonephric canal. During fetal growth, the persistence of the ureteral meatus orifice on the mesonephric canal leads to its anastomosis out of the bladder [2].

It is an underdiagnosed pathology in the male population because it is often asymptomatic. It is often associated with renal dysplasia. In men, the most frequent site of anastomosis is the prostatic urethra. The insertion in the genital tract can occur in seminal vesicles or ejaculatory ducts [3].

Revealing circumstances are dysuria, pyuria, recurrent urinary and genital tract infections, and hypogastric pains [4]. In a case reported by Stimac *et al*., the diagnosis was suspected after a renal cyst puncture found spermatozoids [1].

The association of ureteral ectopia with a double excretory system is exceptional. The prevalence of ureteral duplicity is estimated at 0.8% on autopsy series [5].

It can present as a complete or incomplete duplicity, called bifidity. The situation of ureteral meatus is classically reversed to corresponding urinary systems. Thus, the upper system is drained by the ureter whose anastomosis is located below [1]. Some anomalies may be associated with duplicity, mainly vesicoureteral reflux, megaureter, and ureterocele [6].

In our case, the ureteral ectopia involved a complex malformation of the urinary tract: right ureteral duplicity, a right lower system bifidity with an ectopic insertion in the right lobe of the prostate of the ureter of the right upper system, and a left bifidity.

The association of a ureteral duplicity with an ectopic insertion of one of the ureters is an uncommon phenomenon [2]. Degeneration in this situation is reported in some rare cases, particularly in the male population [7]. It often affects one ureter [8]. Some factors may be involved in the predisposition to oncogenesis, such as urinary stasis and reflux, recurrent infections, and lithogenesis [9]. A case reported by Rao *et al*. described an enteric adenocarcinoma, known for its association with chronic ureteral obstruction [10].

Hematuria is not always the revealing sign, since the ureter can be totally obstructed in its insertion [3].

A CT scan can be limited when it concerns a non-functional kidney or renal dysgenesis. A uro-MRI is more efficient in this case, allowing, in particular, an exact description of the path of the ureter and its distal insertion before surgery. Exploration by an endorectal Doppler can refine the anatomical description of the ureter’s termination [11].

The surgical challenge is summed up in two essential points: the progressive release of the ureter, especially in its pelvic terminal portion, and the preservation of blood supply draining the adjacent urinary system. In fact, the two ureters share the same vascularization. An extensive dissection of the tumorous ureter can compromise the vascularization of the tumor-free ureter [6]. Hemi-nephro-ureterectomy is the most reported technique [8]. It is mandatory to follow the trajectory of the ureter to its termination. A radical prostatectomy associated with a hemi-nephro-ureterectomy was practiced in two cases previously, including one by laparoscopic transperitoneal approach for an ectopic anastomosis in the prostatic urethra [7, 11].

In our case, considering the tumorous ureter anastomosis in the prostatic right lobe, and the absence of an ureteral orifice in the prostatic urethra in endoscopy, the surgical procedure was limited to a right hemi-nephro-ureterectomy without associated radical prostatectomy.

## Conclusions

Ureteral duplicity with ectopic ureteral meatus is an exceptional situation. Symptoms are not specific. Urinary stasis is a predisposing factor to oncogenesis. Even if malignant transformation is very rare, hematuria, especially with risk factors of urothelial tumors, justifies endoscopic and radiological explorations, including uro-MRI, to rule out malignancy.
